# 

**Published:** 1852-04

**Authors:** 


### CONTENTS.

PAGE.
ARTICLE I.—Treatment of Dental Caries, Complicated with Disorders of the Pulp and Peridental Membrane. By Robert Arthur, D. D. S. . . . . . . . . . . . 337
ARTICLE II.—Address Delivered before the Graduating Class of the Baltimore College of Dental Surgery, March 1st, 1852. By Eleazar Parmly, . . . . . . . . . . 353
ARTICLE III.—On Excessive Hemorrhage from the Extraction of Teeth, with Cases. By J. L. Levison, Brighton England, 367
ARTICLE IV.—Review of Dr. Leslie's Report of the Proceedings of the last Meeting of the Mississippi Valley Association of Dental Surgeons. By James Taylor, M. D. D. D. S. Cincinnati, 381
ARTICLE V.—A Historical Sketch of American Dental Patents: with Remarks. By Geo. W. Kendall, . . . . . . 397
ARTICLE VI.—On the Treatment of Deep-seated Dental Caries. By Wm. H. Dwinelle, M. D., D. D. S., Cazenovia, N. Y. 442

SELECTED ARTICLES.

ARTICLE VII.—Teeth—Comparative Anatomy, . . . . 439

ARTICLE VIII.—Queen's Hospital, Birmingham. Reported by W. J Moore, Esq., House-Surgeon, . . . . . . 489

ARTICLE IX—Salivary Fistula, . . . . . . . . 492

ARTICLE X.—Epulis, . . . . . . . . . 493

ARTICLE XI.—Spontaneous Collapse of the Walls of the Antrum, 495

ARTICLE XII.—On Anchylosis of the Lower Jaw. By Dr. Wernher, . . . . . . . . . . . . . 496

BIBLIOGRAPHICAL.

The Half-Yearly Abstract of the Medical Sciences. By G. W. H. Rankin, M. D. . . . . . . . . . . 498

Meigs' Velpeau's Midwifery. By W. Byrd Page, M. D. . 498

The Medical Student's Vade Mecum. By George Mendenhall, M. D. 499

Discourses delivered before the Cincinnati Medical Library Association. By Daniel Drake, M. D. . . . . . . 499

Review of Materia Medica for the use of Students. By John Biddle, M. D. . . . . . . . . . . 499

The Pocket Formulary. By Henry Beasley, . . . . . 499

Ten Minutes Advice respecting the Human Teeth. By Horace Norton, M. D. . . . . . . . . . . . 500

The importance of Preserving the Teeth. By John Linn, Dentist, 500

EDITORIAL DEPARTMENT.

Annual Commencement of the Baltimore College of Dental Surgery, 500
Dr. Trenor and Dental Colleges, . . . . . . . . 501
Allen's Silicious Cement, . . . . . . . . . 502
A New Base for Artificial Teeth, . . . . . . . . 503
Hunter's Silicious Cement, . . . . . . . . . . 503
Fees for Dental Operations Reduced, . . . . . . . 504
Dr. Dumont, . . . . . . . . . . . . . 504
Mechanical Dentistry, . . . . . . . . . . 504

